# Live-Cell Imaging and Analysis Shed Light on the Complexity and Dynamics of Antimicrobial Peptide Action

**DOI:** 10.3389/fimmu.2012.00248

**Published:** 2012-08-14

**Authors:** Alberto Muñoz, Nick D. Read

**Affiliations:** ^1^Fungal Cell Biology Group, Institute of Cell Biology, University of EdinburghEdinburgh, UK

Fungi and bacteria cause many serious and sometimes devastating diseases in humans, animals, and crops. Furthermore, resistance against antimicrobial drugs is steadily increasing. There is thus an urgent need to discover new antimicrobial drugs and drug targets. Antimicrobial peptides (AMPs) are widespread in nature and produced naturally by animals, plants, fungi, and bacteria (Zasloff, 2002). These secreted peptides typically act as broad spectrum antibiotics as part of the innate immune systems of these organisms. AMPs that possess high activity against microbial pathogens are attracting great interest for use as novel therapeutic agents to prevent and treat microbial diseases (Brogden, 2005; Hancock and Sahl, 2006). The cell-penetrating properties of many AMPs facilitate them reaching intracellular targets which, in most cases, are unknown and may be novel (Marcos and Gandía, 2009; Nicolas, 2009). Numerous candidate AMP-based drugs for use in humans, animals, and crops will undoubtedly appear over the next decade. A mechanistic understanding of their mode-of-action will be essential to underpin their use as new antimicrobial drugs, to identify novel drug microbial targets, and assist the rational design of more powerful and specific AMPs and peptidomimetics.

## Live-Cell Imaging of Individual Cells

Live-cell imaging techniques have become powerful tools for understanding the dynamic modes-of-action of AMPs. They complement methods in which AMP- or peptoid-treated cells are typically fixed and then processed for immunolocalization (e.g., Theis et al., 2005), electron microscopy (e.g., Friedrich et al., 2000), atomic force microscopy (e.g., Alves et al., 2010), or X-ray tomography (Uchida et al., 2009).

Live-cell imaging has been used primarily to analyze the effects of AMPs on the morphology and growth of bacteria and fungi. Fluorescent dyes have sometimes been used in these studies (e.g., as reporters of plasma membrane permeabilization, cell death, or to label cell walls). However, most of these studies have been restricted to imaging cells at a specific time point after treatment rather than performing time-lapse imaging and measurements on the same cells to monitor their dynamic changes in response to AMPs. Nevertheless, these studies provide useful information relating to the effects of AMPs on microbial cells, including thickening or weakening of cell walls, cell enlargement/shrinkage, or alterations in cell growth/branching patterns, as well as cell permeabilization and killing. These responses can be related to the stress the cell is sensing but also as defense responses to counteract peptide action. Most studies using live-cell imaging and AMPs have been done with fungi rather than bacteria, due, in part, to the advantages of fungal cells (larger cells, easy visualization, and non-motile). Morphological studies of the effects of plant defensins on fungi, for instance, have resulted in these AMPs being divided into two different subgroups, referred to as *morphogenic* and *non-morphogenic*, according to the type of morphological changes they induce in defensin-sensitive fungi (Thomma et al., 2002). Morphogenic defensins inhibit hyphal growth with a concomitant increase in hyphal branching, whereas non-morphogenic defensins inhibit growth without causing marked changes in cell morphology (Terras et al., 1992; Broekaert et al., 1995). Recently, the γ-core motif within the related *Medicago* defensins MsDef1 and MtDef4 has been shown to contain the major determinants which contribute to their morphogenicity and antifungal activity against the phytopathogenic fungus *Fusarium graminearum* (Sagaram et al., 2011). Another plant defensin, RsAFP2, has been shown to induce septin mislocalization and to impair the yeast-to-hypha transition in the human pathogen *Candida albicans* (Thevissen et al., 2012). In the phytopathogen *Penicillium digitatum*, we have reported alterations in cell morphology, conidiophore formation, and cell wall structure following exposure to the rationally designed peptide PAF26 (Muñoz et al., 2006) and cationic Lactoferricin-derived peptides (Muñoz and Marcos, 2006). Here it was shown, using the chitin-binding fluorophore calcofluor white with the membrane permeabilization reporter dye Sytox Green, that the peptides when used at sub-inhibitory concentrations caused abnormalities in cell morphology and growth pattern without permeabilizing the plasma membrane.

The real innovation for visualizing the dynamics of AMP–microbe interactions in recent years has been achieved with the use of fluorescently labeled peptides (or peptidomimetics) in combination with live-cell imaging, particularly using confocal microscopy. Conveniently, both natural and synthetic AMPs can be fluorescently labeled using commercially available protein tagging protocols (e.g., Lobo et al., 2007; van der Weerden et al., 2008). AMPs can also be chemically synthesized with fluorescent labels (e.g., fluorescein-, rhodamine-, BODIPY-, or Alexa fluor-based dyes, or quantum dots) with the fluorescent group at the N or C terminus of the peptide or peptidomimetic (e.g., Muñoz et al., 2006, 2012; Mochon and Liu, 2008; Jang et al., 2010; Mania et al., 2010; Srinivas et al., 2010). The fluorescent AMPs commonly retain their antifungal activity although this may be reduced, often minimally, compared with the unlabelled AMPs. Most importantly, is that these fluorescent AMPs can be directly observed by live-cell imaging in time course experiments. The use of these AMP conjugates is greatly increasing our knowledge of their interaction, method of penetration, pathways of intracellular transport, and sites of antimicrobial action within living microorganisms. Furthermore, live-cell imaging gives an improved understanding of how the dynamic intracellular localization of the peptide influences the dynamic morphogenesis and physiology of individual microbial cells.

Various antifungal peptides and plant defensins have been shown to be internalized by living fungal cells (Oberparleiter et al., 2003; Theis et al., 2003; Moreno et al., 2006; van der Weerden et al., 2008; Mania et al., 2010; Binder et al., 2011; Maurya et al., 2011). For instance, the antifungal protein AFP from *Aspergillus giganteus* and the plant defensin Psd1 exhibited co-localization with Sytox Green- and DAPI-stained fungal nuclei, respectively (Moreno et al., 2006; Lobo et al., 2007). Nevertheless, in all these examples the mechanism of AMP internalization or their intracellular targets remain unknown. Histatin-5 is an antifungal AMP whose internalization mechanism coupled with activity has been studied in most detail in the pathogen *C*. *albicans*. Two distinct pathways for its intracellular trafficking have been postulated: slower endocytic internalization and a more rapid energy-independent uptake into the cytoplasm and vacuole (Mochon and Liu, 2008; Jang et al., 2010; Jang and Edgerton, 2012). In parallel, we have recently described a concentration-dependent mechanism of cell penetration and killing by the *de novo* designed hexapeptide PAF26 in the fungus *Neurospora crassa* (Muñoz et al., 2012). This peptide was shown to be endocytically internalized at low fungicidal concentrations, accumulating in vacuoles that expanded, and then was actively transported into the cytoplasm, which coincided with cell death. This study used a combination of vital fluorescent dyes (the membrane selective dye, FM4-64; the vacuolar dye, cDFFDA, and the cell death reporter propidium iodide), nuclei labeled with GFP, and either FITC- or TMR-labeled PAF26. These analyses demonstrated the advantages of how the direct observation of a peptide can be related to its subcellular effects at different stages in its antimicrobial action in individual cells. Even though the synthetic PAF26 and natural histatin-5 are cationic peptides, they are structurally unrelated (e.g., PAF26 is a hexapeptide and histatin-5 possesses 24 amino acids), they nevertheless seem to exhibit similar concentration-dependent pathways of internalization. As a result, we have proposed that PAF26 may be used as a simple model for mode-of-action studies of cationic antifungal AMPs such as histatin-5, as well as understanding how different aspects of its activity (e.g., endocytic and passive internalization, intracellular trafficking, and cell killing) are determined by individual residues or domains within its six amino acid sequence (Muñoz et al., 2012). Live-cell imaging and analytical techniques will provide novel insights into these processes.

Fluorescently labeled AMPs have only been used in a few live-cell imaging studies on bacteria. An interesting recent study by Sochacki et al., (2011) showed the dynamic killing by the human AMP LL-37 of single *Escherichia coli* cells using time-lapse imaging. Rhodamine labeled LL-37 was monitored in combination with periplasmic GFP and the dye Sytox green to demonstrate that disruption of the cytoplasmic membrane by the peptide was not the growth-inhibiting mechanism, but rather this was caused by translocation across the outer membrane and access of the peptide into the periplasmic space. Leptihn et al. (2009) investigated the mode-of-action of the S1 peptide using fluorescence correlation spectroscopy (FCS) and single molecule tracking using quantum-dot labeled peptide. Using this approach they elucidated a temporal and spatial perspective of the bactericidal events involved in S1 peptide action.

## Live-Cell Analysis of Cell Populations

The use of live-cell probes to measure and analyze various physiological parameters in AMP-treated populations of living cells has also proven very useful. For these studies, multiwell plate fluorimetry/luminometry or flow cytometry have been used. 96- or 384-microtiter well plate assays provide average measurements across a whole cell population. They are well suited for high throughput analysis and for monitoring changes in various physiological parameters [e.g., membrane potential and permeability, reactive oxygen species (ROS), intracellular calcium] with high temporal resolution. Flow cytometry, on the other hand, is able to generate one or more fluorescence measurements of a physiological or other cell parameter at a single time point for each cell in population. It is thus not suited for dynamic measurements of cell physiology but provides detailed information on the heterogeneity of responses within a cell population. Potentiometric dyes have been used to measure the plasma membrane potential in response to treatment with plant defensins (Thevissen et al., 1996), the protein PAF from *Penicillium chrysogenum* (Leiter et al., 2005), histatin-5 (Helmerhorst et al., 2001b), or PAF26 (Muñoz et al., 2012). Fluorescent dyes have been employed to detect ROS formation following treatment with histatin-5 (Helmerhorst et al., 2001a), VS2 and VS3 (Maurya et al., 2011), PAF26 (Carmona et al., 2012), PAF protein (Leiter et al., 2005), or Lactoferrin (Andrés et al., 2008). The genetically encoded calcium-sensitive, bioluminescent protein aequorin has been used to measure changes in intracellular calcium in response to treatment with the PAF protein (Binder et al., 2010, 2011) or PAF26 (Muñoz et al., 2012). Kim and Cha (2006) used a Förster resonance energy transfer (FRET)-based assay to quantify AMP-induced membrane disruption in *E. coli* by measuring changes in the FRET efficiency of a cytosolic protein when it became released into the lower pH environment of the external medium.

## Future Prospects

The potential of using fluorescent labeled AMPs in combination with multiwell plate measurements or flow cytometry has been explored to only a limit extent. For example, Benincasa et al. (2009) used flow cytometry to distinguish an AMP that was internalized by bacterial cells from another that was membrane active by using cell impermeant trypan blue to quench the fluorescence of fluorescently labeled AMP that was on the cell surface. In the future, the use of “smart probes” to label AMPs will be useful where the fluorescence spectral characteristics of the label changes depending on the cell compartment that the AMP is in. For example, a label that is pH-sensitive could be used to report the transport of the AMP into an acidic organelle such as a vacuole. The targeting of genetically encoded physiological reporters (e.g., ROS-sensitive, pH-sensitive, or calcium-sensitive GFP-based indicators) to different cell compartments or organelles will also be extremely useful to monitor the effects of AMPs on cell populations using multiwell plate fluorimetry or flow cytometry.

There are also now a wide range of advanced, live-cell imaging technologies which are commercially available and that need to be explored with regard to analyzing the influence of fluorescently labeled AMPs on living cells. These include: fluorescence lifetime imaging microscopy and/or FRET microscopy to image and measure interactions between fluorescently labeled AMPs and other molecules (Sekar and Periasamy, 2003; Becker, 2012); fluorescence recovery after photobleaching (FRAP) to visualize and measure processes such as the rate of AMP diffusion or trafficking in cells (Lippincott-Schwartz et al., 2003); FCS to measure binding constants between peptides and other molecules (Bacia and Schwille, 2007); various super-resolution microscopic techniques that allow the spatial resolution achievable with fluorescence microscopy to be significantly increased with living cells (Chi, 2009); and high content, multiparameter imaging which are designed for ultra high throughput analysis of living cells in multiwell plates (Taylor and Haskins, 2007).

In summary, live-cell imaging and analytical techniques are extremely powerful methods that can provide direct, high spatiotemporal resolution information on the dynamics and complexity of the modes-of-action of AMPs, and also novel peptidomimetics (Scorciapino and Rinaldi, 2012). In the future, these approaches will undoubtedly have a profound impact on our understanding of the ways in which AMPs work which should greatly assist the rational design of new and more effective antimicrobial drugs.
